# Counterparts

**Published:** 1887-07-02

**Authors:** 


### COUNTERPARTS.

### BY A STUDENT OF EASTERN PHILOSOPHIES.

*(Continued from p. 212, vol. ii.)*

### CHAPTER IV.—A DISCOVERY.

SHE loved him ! The fatal truth had dawned upon her at last. Her whole being was filled with the rapture of the thought, and in the first few moments of intense, conscious happiness, everything else was forgotten.

Yes ; she loved him, with a depth and fervour of emotion of which she had hitherto believed herself incapable. She felt like a new creature, and already looked back on her old self with wondering pity. She understood now for the first time why she had been so happy during the past three weeks. Why had he left her in the moment of her awakening, when her whole soul was thirsting for his presence ?

"You have plighted your troth to another, and the destiny of both of us is marred."

Edith rose from her chair with a scared look on her face ; it was almost as if she had heard the words uttered aloud, so distinctly did they recur to her mind.

"What shall I do ! what shall I do !" she moaned ; and amid the tumult of distracting thoughts which beset her, there was no answer to her question. Her position in all its terror rose before her. She must either cruelly cast aside the love of a man who, whatever his faults, had been willing to sacrifice his own prejudices and incur the displeasure of a much-adored mother for her sake; or she must—

But the alternative was too horrible to think of. Death would be better than that.

"I shall go mad," she exclaimed, pressing her hands to her head. "I feel as if my brain were on fire, as if I could not breathe."

At this moment Edith caught sight of her aunt's bonnet coming round the corner of the garden wall. Feeling it impossible to speak to anyone in the frame of mind in which she then was, she hastened to her room, flung on her hat, fled out at the back door, and was soon hurrying along the edge of the sea as fast as her feet could carry her.

The ocean was already crimson with the light of the setting sun as Edith wended her way along the shore back to Westborough. She had succeeded in walking off the excitement into which the events of the day had thrown her, and a dull depression of spirits had taken its place. No longer was she torn by conflicting doubts and fears, for she had made up her mind to the worst, having persuaded herself, somewhat illogically, that the most self-sacrificing course must of necessity be the right one ; and the calmness of despair had settled upon her. Her only comfort was in the thought that it could only last for a short time. She was firmly convinced that she could not live long as the wife of any other man than Paul, and death at the present moment had no terrors for her.

"Oh, if you please, Miss Charteris," said a little voice behind her.

Edith turned and stared in an absent-minded way at the little ragged boy who had addressed her.

"If you please, miss, Annie is took ill, and mother is out, and I don't know what to do," said the little fellow, with a piteous quiver of the lip.

"Oh, it is you, Tommy," said Edith, recognising one of her small pensioners. "Annie is ill, did you say ? I will go in and see her."

And, oblivious of the fact that it was long past her dinner-hour, and that she was weak and faint from agitation and want of food, Edith followed the boy into his poor little home, where she found his sister more seriously ill than she expected.

"You run for the doctor, Tommy," she said, as she took the shivering, moaning child in her arms, and began to pet and soothe her.

"What a pity Paul is not here !" she thought, as she looked at the little pain-drawn face on her breast.

But when the doctor came he took the child away from her without ceremony, and told her severely that she had no business there, especially in the weary condition in which she seemed to be.

"If I am not mistaken, the child has got that non-eruptive form of typhus fever which is so prevalent in the neighbourhood now," he remarked testily. "Young ladies should really be more careful."

"I never take fevers," said Edith.

"Humph ! no reason why you should not take them some day," said the doctor, as he tore a leaf from his pocket-book and scribbled some hieroglyphics on it.

"Get this at the chemist's," he said ; "drink it as soon as you get home ; change all your clothes, and have them fumigated at once. And remember, you are not to come here again."

"I am going back to London to morrow," said Edith.

Of course, old Miss Charteris protested indignantly when her niece informed her of her resolve to depart ; but Edith held to her point.

"You know I only came for three weeks, auntie, and I have stayed six. Of course, I don't like leaving you : I shall miss you dreadfully ; but mamma wishes me back again, and I am longing see papa—and Archie."

Miss Charteris grunted sardonically, "Archie, indeed ! Do you know, Edith, I thought you had gone off and married Mr. Travers, when you were two hours late for dinner yesterday."

"No wonder you looked disappointed when I came in," said her niece, with a somewhat dreary smile.

"I don't like letting you go home with that white face, child. You have lost all your roses since yesterday," continued Miss Charteris with concern.

"They will come [back when my headache is better," said Edith.

But her headache got worse instead of better, and by the time she reached London her fear that she was going to be ill had risen to a certainty. The fact did not trouble her much, however ; indeed, she could not help feeling slightly surprised at her own utter indifference to everything. She did not know that this dull apathy was but one of the symptoms of the disease by which she had been stricken.

Days passed. Under the directions of Dr. Owlett, a physician of the old-fashioned kind, who believed in sweating the fever out of a patient, Edith had been placed on a feather bed, a mountain of blankets had been piled upon her, her room made as air-tight as possible, and now she was raving in delirium.

"Raving, surely," thought poor Archie, as he stood outside her door and heard her implore her mother to "take Archie away and let Paul come to her."

Mrs. Charteris had in vain tried to keep him downstairs out of hearing. Archie unfortunately had not the same dread of infection that she had. Good Mrs. Charteris did her duty as a mother, and nursed her daughter herself; but she always did so with a handkerchief steeped in camphor held firmly between her teeth.

"He is trying to come in, and they won't let him. Mother, I must go to him," cried Edith, making frantic efforts to get out of bed, which were, of course, frustrated.

There was an ugly look on Archie's face as he went downstairs and entered one of the front rooms.

"I would rather see her die than let him come near her," was his uppermost thought; and as it lay on his mind there drove up to the door a hansom, out of which stepped Paul Travers.

A dark look of hate swept across Archie's face as he went into the hall to meet Bridget, the maid-of-all-work, who was coming up from the area to answer the bell.

"Bridget," said Archie, "don't open the door, but look through the letter-hole at the man on the steps."

As this was a constant practice of Bridget's, she had no scruples in complying with the command, and contemplated Paul's back for some moments through the hole.

"Now, Bridget," continued Archie, "I want you to keep that man out of the house. He has the evil eye, and will work harm to you, and Miss Edith, and everybody here, if he once gets inside. I believe"—here Archie sank his voice to a mysterious whisper, which made Bridget's flesh creep—"I believe he has *sold his soul to the devil!*"

"Mercy on us!" exclaimed Bridget, crossing herself.

"So you must keep the chain on the door, and never open it to anybody, if he is anywhere near; and, Bridget, here is a sovereign for you, and I will give you another if you succeed in keeping that fellow out of the house."

(*To be concluded.*)

